# Response rate and factors associated with response in patients with schizophrenia undergoing bilateral electroconvulsive therapy

**DOI:** 10.1192/bjo.2023.37

**Published:** 2023-04-24

**Authors:** Chanaichon Ruangsetakit, Pichai Ittasakul

**Affiliations:** Department of Psychiatry, Faculty of Medicine, Ramathibodi Hospital, Mahidol University, Bangkok, Thailand

**Keywords:** Acute ECT, electroconvulsive therapy, in-patient, response rate, schizophrenia

## Abstract

**Background:**

Schizophrenia is a severe mental illness and a common indication for electroconvulsive therapy (ECT). Research is lacking on the factors that influence response to acute ECT treatment in schizophrenia patients.

**Aims:**

This study examined the response rate and associated factors in patients with schizophrenia undergoing bilateral ECT.

**Method:**

Demographic data, clinical characteristics, ECT data and treatment response were respectively reviewed in patients with schizophrenia undergoing bilateral ECT from January 2013 to June 2022.

**Results:**

Forty-six patients were included. Nine responded after the first three sessions, 17 after six sessions, 20 after nine sessions, 25 after 12 sessions and 28 after the last ECT session, cumulatively. The mean of the baseline Brief Psychiatric Rating Scale psychotic symptom subscale score was significantly higher in responders (17.0) than non-responders (10.9) (*P* < 0.05). The mean of duration of electroencephalogram seizure was significantly longer in responders (53.9) than in non-responders (42.7). There was no association between demographic and ECT data and treatment response. Among 28 responders, 20 responded to ECT after nine sessions (faster responders) and eight responded later (slower responders). The number of failed antipsychotics prior to ECT was 2.8 for faster responders and 4.4 for slower responders (*P* = 0.02). Nominal logistic regression showed that the number of failed antipsychotics prior to ECT was associated with speed of response to ECT (*P* = 0.037, odds ratio = 1.77).

**Conclusions:**

ECT is an effective treatment for schizophrenia and may be influenced by the number of failed antipsychotics prior to ECT.

Schizophrenia is a chronic psychiatric disorder and one of the most severe and disabling mental illnesses. Globally, around 24 million people, or 1 in 300 persons (0.32%), suffer from schizophrenia.^1^ The prevalence of schizophrenia in Thailand is 8.8 per 1000.^2^ Approximately 30% of patients respond poorly to antipsychotic treatment.^3^ Treatment-resistant schizophrenia has a poor prognosis owing to long-term loss of function and symptom recurrence.

Electroconvulsive therapy (ECT) was originally developed for the treatment of psychosis and remains an important treatment for schizophrenia. Studies have shown that ECT is an effective treatment for enhancing therapeutic efficacy in patients with schizophrenia, especially those who do not respond to antipsychotic drugs.^4–7^ Estimated rates of remission for schizophrenia patients treated with ECT range from 40–80%,^8–11^ with a likely response in patients with catatonia or prominent mood symptoms.^12,13^ A recent study demonstrated that ECT improved symptoms and increased the rate of discharge for hospital-admitted patients with schizophrenia compared with medication alone or no ECT.^4^

Worldwide, schizophrenia may be the most common indication for ECT.^14,15^ In our institution (Ramathibodi Hospital), schizophrenia is also the most common diagnosis of all patients treated with ECT.^16,17^ Some studies have investigated predictors of treatment response to ECT in patients with schizophrenia. They showed that the factors that were associated with treatment response were long-acting injectable antipsychotics, comorbid substance use, absence of treatment with antiepileptic medication, a previous good response to ECT and primary indication for ECT referral other than failed pharmacotherapy.^10,11^ However, few studies have focused on the factors influencing response to acute treatment in patients with schizophrenia. To fill this gap, in this study, we examined response rate, factors associated with response and speed of response in patients with schizophrenia undergoing bilateral ECT.

## Method

### Setting and study design

The study protocol was approved by the Ethics Committee on Human Experimentation of the Faculty of Medicine, Ramathibodi Hospital, Mahidol University, Bangkok, Thailand (COA. MURA2022/214). Written informed consent was obtained from all patients.

The research was conducted as a retrospective study. All patients with schizophrenia undergoing in-patient ECT at Ramathibodi Hospital, Bangkok, Thailand, from January 2013 to June 2022 were reviewed. Diagnosis was performed by psychiatrists using criteria from the DSM-5.^18^ The severity of psychiatric symptoms was assessed by psychiatrists and residents-in-training using the Brief Psychiatric Rating Scale (BPRS) score.^19–21^ Interrater reliability was assessed using the intraclass correlation coefficient (ICC). BPRS received an ICC of 0.9.

Prior to receiving ECT, all patients were assessed by psychiatrists and anaesthesiologists. At least 15 h before treatment, benzodiazepines were discontinued. The ECT procedure was performed at the post-anaesthesia care unit. The ECT team included a psychiatrist, in-training psychiatric residents, anaesthetic staff, psychiatric nurses and anaesthetic nurses. Following anaesthesia with thiopental (1.5–2.5 mg/kg intravenous [i.v.]) or propofol (1–2 mg/kg i.v.) and a muscle relaxant (succinylcholine, 0.5–1.5 mg/kg i.v.), ECT was given. The procedure used a modified technique that incorporated a brief pulse wave generated by a Mecta Spectrum 5000Q (Mecta, Tualatin, OR, USA) or Thymatron System IV (Somatics, Northampton, MA, USA).^22^ All patients with schizophrenia had ECT with bilateral electrode placement and a pulse width of 0.5–1.0 ms.

During the first ECT session, dose titration was used to determine the seizure threshold. The dose titration schedule and parameter settings for ECT devices are shown in Table 1 of the Supplementary Material. For subsequent ECT sessions, the stimulus intensity was raised to 50% above the seizure threshold.^23^ ECT was administered three times per week.

**Table 1.** Demographic data and clinical characteristics (*n* = 46)

Clinical characteristics	Mean ± s.d. or *n* (%)			χ^2^	*t*	*P*-value
	All patients (*n* = 46)	Non-responders (*n* = 18)	Responders (*n* = 28)			
Age, years	43.7 ± 12.6	44.4 ± 11.9	43.3 ± 13.2		0.29	0.77
Age at onset, years	26.1 ± 8.5	25.5 ± 7.6	26.5 ± 9.2		−0.39	0.70
Duration of illness, years	17.4 ± 8.8	18.7 ± 8.6	16.6 ± 9.1		0.77	0.44
Gender						
Female	24 (52.2%)	11 (61.6%)	13 (46.4%)	0.95		0.33
Male	22 (47.8%)	7 (38.9%)	15 (53.6%)			
History of previous ECT						
No	18 (39.1%)	8 (44.4%)	10 (35.7%)	0.35		0.55
Yes	28 (60.9%)	10 (55.6%)	18 (64.3%)			
Number of concurrent antipsychotic medications	2.0 ± 1.0	2.1 ± 1.1	1.9 ± 0.9		0.75	0.46
Number of failed antipsychotics prior to ECT	3.5 ± 1.9	3.3 ± 1.8	3.7 ± 1.9		−0.71	0.48
<2 medications	6 (13.0%)	3 (16.7%)	3 (10.7%)	0.34		0.67
≥2 medications	40 (87.0%)	15 (83.3%)	25 (89.3%)			
Total BPRS score						
Before ECT	46.9 ± 19.7	37.3 ± 16.7	53.0 ± 19.2		−2.93	0.006
After last ECT*	26.6 ± 10.8	31.3 ± 15.6	23.5 ± 4.1		2.54	0.02
Total BPRS psychotic symptom subscale score						
Before ECT*	14.7 ± 6.7	10.9 ± 6.4	17.0 ± 5.8		−3.26	0.003
After last ECT*	7.2 ± 4.0	9.1 ± 5.2	6.0 ± 2.5		2.33	0.03

BPRS, Brief Psychiatric Rating Scale; ECT, electroconvulsive therapy.

* *P* < 0.05.

### Data collection

Demographic data, clinical characteristics of patients and ECT data were obtained.

#### Demographic data and clinical characteristics

Participants’ data were reviewed, including age, gender, age at onset, duration of illness, history of previous ECT, number of concurrent antipsychotic medications, number of failed antipsychotics prior to ECT, and severity of psychotic symptoms before and after ECT.

#### ECT data and treatment response

ECT data of participants were collected, including reasons for ECT, maximum charge, motor and electroencephalogram (EEG) seizure durations, postictal suppression index and total number of ECT sessions.

Treatment response was defined as a 40% reduction on the BPRS psychotic symptom subscale (hallucinatory behaviour, suspiciousness, conceptual disorganisation and unusual thought content) from pre-treatment scores to last ECT treatment scores.^4,24^ Among responders, we divided patients into faster responders (ECT ≤9 sessions) and slower responders (ECT >9 sessions).

### Statistical analysis

Numbers and percentages of patients were used to summarise nominal data, such as gender and history of ECT. Based on normality of distribution, continuous variables (such as age) were summarised as the mean with standard deviation. For categorical variables, chi-squared test or Fisher's test was used for analysis. For continuous variables, an independent *t*-test was used.

The clinical characteristics of patients and clinical outcomes were analysed to determine factors associated with treatment response. Associations among factors were examined using multivariable logistic regression. Among responders, subgroup analysis was performed by nominal logistic regression to determine the factors associated with speed of response to ECT.

All statistical analyses were performed using SPSS 26.0 for Windows (IBM Corp., Armonk, NY, USA). *P*-values less than 0.005 were considered to indicate statistical significance.

## Results

From January 2013 to June 2022, 58 patients with schizophrenia received in-patient ECT as an acute treatment. Twelve (20.7%) patients were excluded owing to missing data and 46 (79.3%) were included for analysis. The reasons for undertaking ECT were: non-response to antipsychotics (27, 58.7%), agitation (11, 23.9%), psychomotor retardation (5, 10.9%) and intolerance to side-effects of psychotropic medication (3, 6.5%). The mean (s.d.) total number of ECT sessions was 12.9 (5.8) (range: 5–28).

### Non-responders versus responders

**Fig. 1:**
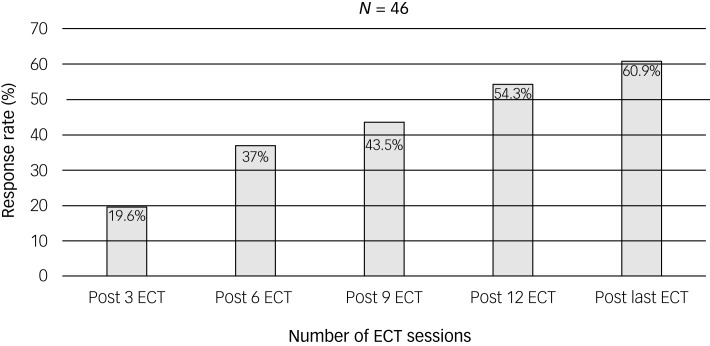
Number of electroconvulsive therapy sessions and response rate.

After receiving ECT, 28 of 46 patients (60.9%) were responders (defined by a >40% reduction on the BPRS psychotic symptom subscale) and 18 (39.1%) were non-responders. Altogether, nine (19.6%) responded after the first three sessions, 17 (37.0%) after six sessions, 20 (43.5%) after nine sessions, 25 (54.3%) after 12 sessions and 28 (60.9%) by the last ECT session (Fig. 1). The demographic data, ECT data, and clinical characteristics of all participants are shown in Tables 1 and 2.

**Table 2:** Comparison of ECT data between non-responders and responders (*n* = 46)

Clinical characteristics	Mean ± s.d. or *n* (%)		χ^2^	*t*	*P*-value
	Non-responders (*n* = 18)	Responders (*n* = 28)			
Reasons for ECT					
Non-response to antipsychotics	9 (50.0%)	18 (64.3%)	0.92		0.34
Agitation	6 (33.3%)	5 (17.9%)	1.44		0.23
Psychomotor retardation	2 (11.1%)	3 (10.7%)	0.002		1
Intolerance of side-effects	1 (5.9%)	2 (7.1%)	0.045		1
Maximum charge (mC)	365.5 ± 208.5	328.9 ± 170.1		0.65	0.52
Motor seizure (s)	32.3 ± 6.6	35.7 ± 9.1		−1.38	0.18
EEG seizure (s)*	42.7 ± 11.0	53.9 ± 22.6		−2.23	0.03
Postictal suppression index	80.6 ± 12.4	77.9 ± 17.1		0.41	0.68
Total number of ECT sessions	13.8 ± 6.2	12.3 ± 5.4		0.92	0.36

* *P* < 0.05.

The mean (s.d.) baseline BPRS score and BPRS psychotic symptom subscale score were significantly higher in responders [53.0 (19.2) and 17.0 (5.8), respectively] than non-responders [37.3 (16.7) and 10.9 (6.4), respectively].

Regarding ECT data, the mean (s.d.) of duration of EEG seizure was significantly longer in responders [53.9 (22.6)] than non-responders [42.7 (11.0)].

### Factors associated with response to ECT

Multivariable logistic regression was performed to investigate associations between demographic data, ECT data and response to treatment. We found no association between any demographic or ECT variable (Tables 1 and 2) and response to ECT (Table 2 in Supplementary Material).

### Factors associated with speed of response

Among responders, most patients (20 of 28, 71.4%) responded after nine sessions. Thus, we divided patients into faster responders (ECT ≤9 sessions) and slower responders (ECT >9 sessions). Of the 28 patients in the responder group, 12 (42.9%) were faster responders and 16 (57.1%) were slower responders.

Next, we performed subgroup analysis. There was no difference in demographic and ECT data except for the number of failed antipsychotics prior to ECT (Supplementary Tables 3 and 4 available at https://doi.org/10.1192/bjo.2023.37). The mean (s.d.) of the number of failed antipsychotics prior to ECT was 2.8 (1.6) for faster responders and 4.4 (1.9) for slower responders (*t* = −2.48, d.f. = 25.4, *P* = 0.02). A nominal logistic regression was performed. We found that the number of failed antipsychotics prior to ECT was associated with speed of response to ECT (*P* = 0.037, odds ratio = 1.77).

## Discussion

This was a retrospective study examining response rate and the factors associated with the response and speed of response in patients with schizophrenia undergoing bilateral ECT. We found that the most common reason for ECT was non-response to antipsychotic medication, followed by agitation, psychomotor retardation and intolerance of side-effects of psychotropic medication. This may reflect the fact that when antipsychotic medications are ineffective or intolerable, ECT is usually used as a last option. According to a recent study, ECT can be used effectively in a variety of circumstances for patients with schizophrenia; it is not only advantageous for treatment-resistant schizophrenia.^25^ In this study, each individual underwent an average of 12.9 ECT sessions, which is comparable with the number in our earlier study.^17^ After nine ECT sessions, more than half the patients (54.3%) had responded. Nevertheless, the data showed that patients with schizophrenia received a highly variable number of ECT sessions.^26^

The response rate for ECT was 60.9% in this study. This was slightly higher than the response rates reported by earlier studies, specifically, 50%^4,27^ and 54.5%.^28^ This may be explained by the fact that the populations in those earlier trials only included patients with schizophrenia who had not responded to at least two antipsychotic drugs, whereas in our study, we also included patients who received ECT for other indications. Responders had more severe symptoms prior to ECT than non-responders. As a result, the evidence suggests that ECT may be suitable for schizophrenia patients with severe symptoms. We found that duration of EEG seizures was significantly longer in responders than non-responders. However, there was no association between EEG length and response to ECT. This outcome was consistent with a prior study, which demonstrated that clinical improvement of patients with schizophrenia who received ECT was not affected by seizure duration.^29^ In addition, we found no association between demographic characteristics and ECT data or ECT response.

To examine factors associated with speed of response to ECT, we performed subgroup analysis of the responder group using logistic regression. We found that the number of failed antipsychotics prior to ECT was associated with speed of response to ECT. Faster responders had used fewer antipsychotics than slower responders. As a result, patients with fewer failed antipsychotic prescriptions may benefit more from ECT, whereas excessive antipsychotic use is linked to reduced effectiveness of ECT. Studies of patients with bipolar depression and major depressive disorder have demonstrated similar results, showing that patients who were prescribed many medications had a limited response to ECT.^30,31^

A strength of our study is that it is one of the few to have examined response rate and the factors influencing how patients with schizophrenia respond.

In summary, the current study aimed to identify factors that influenced the response of patients with schizophrenia to ECT treatment. Our results show the effectiveness of patients undergoing ECT therapy. The number of failed antipsychotics prior to ECT was associated with speed of response to ECT. Further investigation is needed to search for other factors associated with treatment response in patients with schizophrenia.

### Limitations

Our study had certain limitations. First, the small sample size might have contributed to the lack of significance. Second, the results should be interpreted cautiously in contexts differing from the in-patient condition in a Thai university hospital. Third, because this was a retrospective study, it was prone to recall bias and cannot be used to determine causality. Fourth, the BPRS scores after ECT were from different durations of ECT, and the number of sessions affects treatment response. Last, other factors including subtype of schizophrenia, suicidality, mood disorders, and ECT procedure may have influenced response to ECT and were not measured in the current clinical samples.

## Data Availability

The data presented in this study are available upon request from the corresponding author.

## Supplementary material

For supplementary material accompanying this paper visit http://doi.org/10.1192/bjo.2023.37. click here to view supplementary material
