# Supplementary material for: Response rate and factors associated with response in patients with schizophrenia undergoing bilateral electroconvulsive therapy
Source: BJPsych Open. 2023 Apr 24;9(3):e75. doi: 10.1192/bjo.2023.37 (PMC10134241; doi:10.1192/bjo.2023.37)
Supplement: Supplementary file 1 [file S2056472423000376sup001.docx]

**Supplementary Files**

**Table 1:** Dose titration schedule and parameter settings for ECT devices

|  | Thymatron System IV | | | | Mecta Spectrum 5000Q | | | | |
| --- | --- | --- | --- | --- | --- | --- | --- | --- | --- |
| Step | Pulse width (ms) | Frequency  (Hz) | Energy  Level  (%) | Charge  (mC) | Pulse width (ms) | Frequency  (Hz) | Duration  (sec) | Current  (mA) | Charge  (mC) |
| 1 | 0.5 | 40 | 10 | 50 | 1 | 40 | 0.75 | 800 | 48 |
| 2 | 0.5 | 40 | 15 | 76 | 1 | 40 | 1.25 | 800 | 80 |
| 3 | 0.5 | 40 | 25 | 126 | 1 | 40 | 2.0 | 800 | 128 |
| 4 | 0.5 | 40 | 35 | 176 | 1 | 60 | 2.0 | 800 | 192 |
| 5 | 0.5 | 40 | 50 | 252 | 1 | 60 | 3.0 | 800 | 288 |
| 6 | 0.5 | 60 | 70 | 353 | 1 | 60 | 4.5 | 800 | 432 |
| 7 | 1.0 | 40 | 100 | 504 | 1 | 60 | 6.0 | 800 | 576 |

**Table 2**: Logistic regression results for factors associated with response in patients with schizophrenia undergoing bilateral electroconvulsive therapy

| **Variables** | **Multivariate** | | |
| --- | --- | --- | --- |
|  | **aOR** | **95% CI** | **P-value** |
| Age (years) | 0.71 | 0.33 – 1.53 | 0.39 |
| Age of onset (years) | 1.47 | 0.70 – 3.11 | 0.31 |
| Total number of ECT sessions | 0.92 | 0.78 – 1.08 | 0.29 |
| Duration of illness (years) | 1.31 | 0.60 – 2.88 | 0.50 |
| Gender | 0.25 | 0.05 – 1.38 | 0.11 |
| History of previous ECT | 1.85 | 0.38 – 9.08 | 0.45 |
| Number of concurrent antipsychotic medications | 0.91 | 0.35 – 2.37 | 0.85 |
| Number of failed antipsychotics prior ECT | 1.24 | 0.81 – 1.88 | 0.32 |
| Maximum charge (mC) | 1.00 | 0.98 – 1.02 | 0.70 |
| Mean motor seizure (s) | 1.30 | 0.37 – 4.54 | 0.68 |
| Mean EEG seizure (s) | 0.94 | 0.39 – 2.26 | 0.90 |
| Postictal suppression index | 0.96 | 0.72 – 1.27 | 0.64 |
| Total BPRS score  Before ECT  After last ECT | 0.92  0.44 | 0.60 – 1.41  0.13 – 1.49 | 0.72  0.19 |
| Total BPRS psychotic symptom subscale score    Baseline  After last ECT | 2.96  0.71 | 0.48 - 18.17  0.54 – 0.94 | 0.24  0.02 |

aOR: adjusted Odd Ratios, CI: Confidence Interval

**Table 3:** Demographic data and clinical characteristics between faster responders and slower responders (n=28)

| Clinical characteristics | Mean $\pm$ SD or n (%) | | | χ2 | T | p-value |
| --- | --- | --- | --- | --- | --- | --- |
|  | All responders  (n=28) | Faster responders (n=12) | Slower responders (n=16) |  |  |  |
| Age (years) | 43.3 $\pm$13.2 | 42.1 $\pm$12.9 | 44.2 $\pm$ 13.7 |  | -0.43 | 0.67 |
| Age of onset (years) | 25.7 $\pm$ 8.1 | 27.1 $\pm$ 10.1 | 25.2 $\pm$7.9 |  | -0.41 | 0.69 |
| Duration of illness (years) | 16.6 $\pm$9.1 | 15.8 $\pm$7.3 | 17.2 $\pm$10.4 |  | -0.39 | 0.7 |
| Gender | | | | | | |
| Female  Male | 13 (46.4 %)  15 (53.6%) | 8 (66.7%)  4 (33.3%) | 5 (31.3%)  11 (68.8%) | 3.46 |  | 0.13 |
| History of previous ECT | | | | | | |
| No  Yes | 10 (35.7%)  18 (64.3%) | 6 (50.0%)  6 (50.0%) | 4 (25.0%)  12 (75.0%) | 1.87 |  | 0.13 |
| Number of concurrent antipsychotic medications | 1.9 $\pm$ 0.9 | 1.6 $\pm$ 0.5 | 2.1 $\pm$ 1.0 |  | -1.67 | 0.11 |
| Number of failed antipsychotics prior ECT | 3.7 $\pm$1.9 | 2.8 $\pm$1.6 | 4.4 $\pm$1.9 |  | -2.48 | 0.02 |

**Table 4:** ECT data and clinical characteristics between faster responders and slower responders (n=28)

| Clinical characteristics | Mean $\pm$ SD or n (%) | | | χ2 | t | p-value |
| --- | --- | --- | --- | --- | --- | --- |
|  | All responders  (n=28) | Faster responders (n=12) | Slower responders (n=16) |  |  |  |
| Maximum charge (mC) | 328.9 $\pm$170.1 | 269.6 $\pm$178.9 | 373.3 $\pm$ 153.9 |  | -1.65 | 0.11 |
| Mean motor seizure (s) | 35.7 $\pm$ 9.1 | 37.3 $\pm$ 10.0 | 34.4 $\pm$ 8.4 |  | 0.83 | 0.41 |
| Mean EEG seizure  (s) | 53.9 $\pm$ 22.6 | 62.3 $\pm$ 30.4 | 47.6 $\pm$ 12.0 |  | 1.77 | 0.09 |
| Postictal suppression index | 77.9 $\pm$ 17.1 | 71.6 $\pm$ 28.0 | 80.4 $\pm$ 11.8 |  | -0.86 | 0.41 |
| Total BPRS score |  | | |  |  |  |
| Before ECT  After last ECT | 53.0 $\pm$ 19.2  23.5 $\pm$ 4.1 | 56.8 $\pm$ 23.6  23.8 $\pm$ 4.6 | 50.2 $\pm$ 15.4  23.2 $\pm$ 3.8 |  | 0.89  0.37 | 0.38  0.72 |
| Total BPRS psychotic symptom subscale score |  | | | | | |
| Baseline  After last ECT | 17.0 $\pm$ 5.8  6.0 $\pm$ 2.5 | 19.2 $\pm$ 5.5  5.7 $\pm$ 2.1 | 15.4 $\pm$ 5.7  6.3 $\pm$ 2.8 |  | 1.75  -0.60 | 0.09  0.55 |

n: number, SD; standard deviation, mC: millicoulomb, χ2: Chi-Square
